# Effects of Toxic AGEs (TAGE) on Human Health

**DOI:** 10.3390/cells11142178

**Published:** 2022-07-12

**Authors:** Masayoshi Takeuchi, Akiko Sakasai-Sakai, Takanobu Takata, Jun-ichi Takino, Yoshiki Koriyama

**Affiliations:** 1 Department of Advanced Medicine, Medical Research Institute, Kanazawa Medical University, 1-1 Daigaku, Uchinada-machi, Kahoku 920-0293, Ishikawa, Japan; asakasai@kanazawa-med.ac.jp; 2 Department of Life Science, Medical Research Institute, Kanazawa Medical University, 1-1 Daigaku, Uchinada-machi, Kahoku 920-0293, Ishikawa, Japan; takajjjj@kanazawa-med.ac.jp; 3 Department of Biochemistry, Faculty of Pharmaceutical Sciences, Hiroshima International University, 5-1-1 Hirokoshingai, Kure 737-0112, Hiroshima, Japan; j-takino@hirokoku-u.ac.jp; 4 Graduate School and Faculty of Pharmaceutical Sciences, Suzuka University of Medical Science, 3500-3 Minamitamagaki, Suzuka 513-8670, Mie, Japan; koriyama@suzuka-u.ac.jp

**Keywords:** advanced glycation end-products (AGEs), toxic AGEs (TAGE), lifestyle-related diseases (LSRD), healthy life expectancy, human health

## Abstract

The habitual and excessive consumption of sugar (i.e., sucrose and high-fructose corn syrup, HFCS) is associated with the onset and progression of lifestyle-related diseases (LSRD). Advanced glycation end-products (AGEs) have recently been the focus of research on the factors contributing to LSRD. Approaches that inhibit the effects of AGEs may be used to prevent and/or treat LSRD; however, since the structures of AGEs vary depending on the type of reducing sugars or carbonyl compounds to which they respond, difficulties are associated with verifying that AGEs are an etiological factor. Cytotoxic AGEs derived from glyceraldehyde, a triose intermediate in the metabolism of glucose and fructose, have been implicated in LSRD and are called toxic AGEs (TAGE). A dietary imbalance (the habitual and excessive intake of sucrose, HFCS, or dietary AGEs) promotes the generation/accumulation of TAGE in vivo. Elevated circulating levels of TAGE have been detected in non-diabetics and diabetics, indicating a strong relationship between the generation/accumulation of TAGE in vivo and the onset and progression of LSRD. We herein outline current findings on “TAGE as a new target” for human health.

## 1. Introduction

The chronic consumption of sugar-sweetened beverages (SSB) and processed foods, which have a very high sugar content (i.e., high-fructose corn syrup, HFCS, and sucrose), has been implicated in the development of obesity and metabolic syndrome (MetS) as well as in the onset and progression of diabetes mellitus (DM) and diabetic complications, cardiovascular disease (CVD), non-alcoholic steatohepatitis (NASH), cancer, and Alzheimer’s disease (AD); however, the underlying mechanisms have not yet been clarified in detail [1,2,3,4]. The formation of advanced glycation end-products (AGEs) occurs under hyperglycemic conditions through the Maillard reaction (a non-enzymatic glycation reaction) between proteins/amino acids and reducing sugars [5,6]. The sugars (i.e., glucose, fructose, and glyceraldehyde (GA)) or carbonyl compounds (i.e., glycolaldehyde, glyoxal (GO), methylglyoxal (MGO), and 3-deoxyglucosone (3-DG)) involved in this reaction influence the types of AGEs generated. Marked variations have been reported in the structures of AGEs in vivo, and complex reactions are needed for their formation; therefore, the structures of only a few AGEs have been elucidated [5,6,7,8].

Sugar metabolic pathways (i.e., glycolysis, the polyol pathway, and fructolysis) lead to the production of GA [9,10,11], which, in turn, contributes to the generation of a group of compounds called GA-derived AGEs (GA-AGEs). These AGEs are strongly cytotoxic, and thus are referred to as toxic AGEs (TAGE) [12]. The accumulation of TAGE has been shown to play a role in the pathogenesis of DM and diabetic complications [13,14,15], NASH [16,17,18,19], CVD [20,21,22], AD [23,24,25], and cancer [26,27,28,29]. In a previous review, we summarized, in detail, TAGE accumulation and cell damage in the brain, liver, and heart [11]. In this review, the content that overlaps with the previous paper has been simplified, and novel findings have been added. In our recent study, the intracellular generation/accumulation of TAGE was detected in many cell types [30,31,32,33,34,35,36,37,38,39,40,41,42]. Elevated levels of TAGE damage cells, which results in the leakage of TAGE into the circulation and to the surrounding cells; the damage induced promotes the onset and progression of lifestyle-related diseases (LSRD) [9,10,11]. Extracellular TAGE interact with the receptor for AGEs (RAGE), which affects intracellular signaling, gene expression, and the release of pro-inflammatory molecules; they also increase the generation of reactive oxygen species (ROS) by different cell types [15], all of which have been implicated in the pathological changes associated with LSRD.

The accumulation of ROS under oxidative stress conditions results in the induction of lipid peroxidation and glycoxidation reactions, which leads to the elevated endogenous production of reactive aldehydes and their derivatives, such as GO, MGO, malondialdehyde, and 4-hydroxy-2-nonenal, giving rise to AGEs and advanced lipoxidation end-products (ALEs). AGEs and ALEs play key roles in the cellular response to oxidative stress stimuli through the regulation of a variety of cell signaling pathways [43]. ROS are also important for aging and health promotion, but in this review, we focus on the importance of intracellular TAGE accumulation from the perspective of glycation.

**Figure 1:**
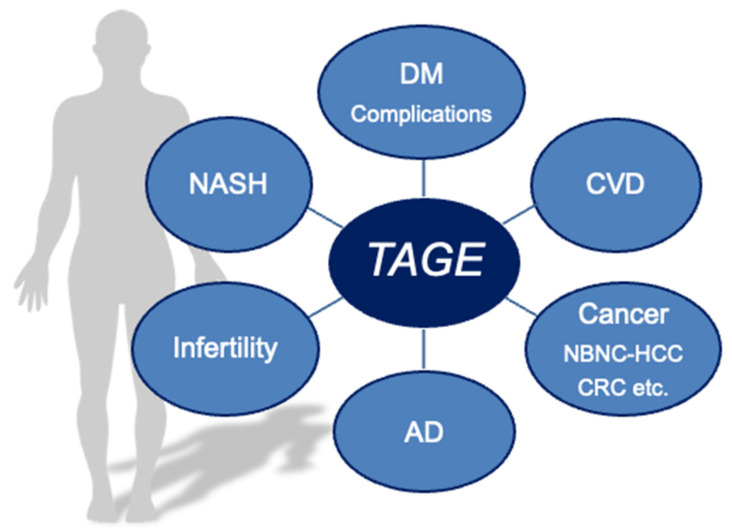
TAGE as a new target for human health. AD: Alzheimer’s disease; CRC: colorectal cancer; CVD: cardiovascular disease; DM: diabetes mellitus; NASH: non-alcoholic steatohepatitis; NBNC-HCC: non-B or non-C (NBNC)-hepatocellular carcinoma; TAGE: toxic advanced glycation end-products.

We herein describe the relationships between intracellular TAGE levels and damage to various cell types. Circulating levels of TAGE have also been shown to contribute to LSRD. The “TAGE theory” is a novel concept that will provide novel insights for research on human health (Figure 1).

## 2. Production of AGEs in the Human Body

**Figure 2:**
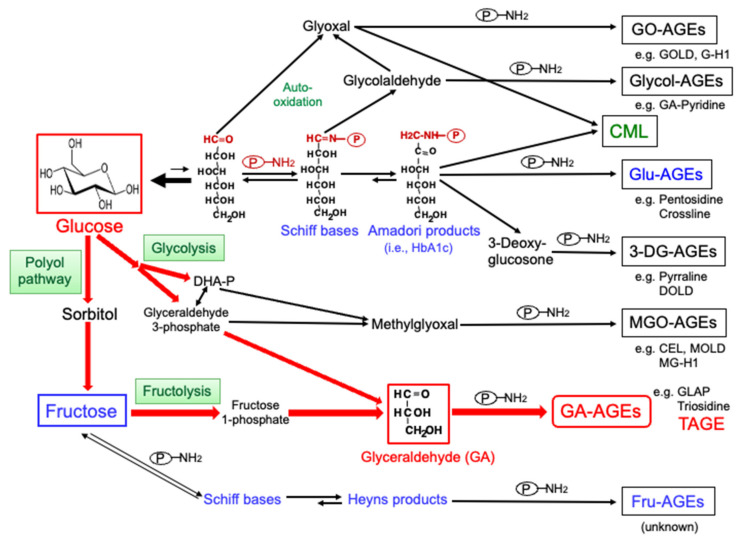
Routes for the production of advanced glycation end-products (AGEs) in the human body. DHA-P: dihydroxyacetone-phosphate; HbA1c: hemoglobin A1c; GO-AGEs: glyoxal (GO)-derived AGEs; Glycol-AGEs: glycolaldehyde-derived AGEs; CML: Nε-(carboxymethyl)lysine; Glu-AGEs: glucose-derived AGEs; MGO-AGEs: methylglyoxal (MGO)-derived AGEs; 3-DG-AGEs: 3-deoxyglucosone (3-DG)-derived AGEs; GA-AGEs: glyceraldehyde (GA)-derived AGEs; Fru-AGEs: fructose-derived AGEs; GOLD: GO-lysine dimer; G-H1: GO-derived hydroimidazolone 1; GA-pyridine: glycolaldehyde-derived pyridine; DOLD: 3-DG-lysine dimer; CEL: Nε-(carboxyethyl)lysine; MOLD: MGO-lysine dimer; MG-H1: MGO-derived hydroimidazolone 1; GLAP: glyceraldehyde-derived pyridinium; TAGE: toxic AGEs; P-NH_2_: free amino residue of a protein.

The production and accumulation of AGEs occurs in tissues with aging, and is accelerated in patients with DM [5,15,44,45]. We previously identified seven distinct classes of AGEs and Nε-(carboxymethyl)lysine (CML) (Figure 2) in the serum of hemodialysis (HD) patients with diabetic nephropathy (HD-DN) [5,46]. Figure 2 shows the production of AGEs through a process involving metabolic pathways for sugar, including sugar auto-oxidation and the Maillard reaction. CML, MGO-derived AGEs (MGO-AGEs), and glucose-derived AGEs (Glu-AGEs) have been the focus of research in the field of LSRD. Serum AGE fractions from HD-DN patients were previously shown to exert neurotoxic effects, which were only neutralized by the addition of the anti-TAGE antibody [23]. In other words, cell damage appears to be induced by TAGE structures that are recognized by the anti-TAGE antibody. TAGE have the strongest binding affinity for RAGE [47,48], with binding inducing the production of ROS by NADPH oxidase, vascular endothelial growth factor (VEGF) expression, and pro-inflammatory cytokine production in many cells [15,49,50], all of which may contribute to the onset and progression of LSRD.

We previously prepared a specific antibody to analyze the toxicity of GA-AGEs [51]. This antibody recognized different epitopes to GA-derived structures, such as 3-hydroxy-5-hydroxymethyl-pyridinium (GLAP) [52] and triosidines [53], but not AGEs with well-characterized CML and Nε-(carboxyethyl)lysine (CEL) or various AGEs (i.e., pyrraline, pentosidine, crossline, argpyrimidine, GO- or MGO-lysine dimers, and GO- or MGO-derived hydroimidazolone) produced from reducing sugars and carbonyl molecules [54,55]. It also did not recognize AGEs with unknown structures, such as GO-/glycolaldehyde-/glucose-/3-DG-/MGO-/GA-/fructose-derived AGEs [51,54,55]. Therefore, unknown GA-AGE structures that are recognized by the anti-TAGE antibody may be cytotoxic. Based on these findings, AGEs that are recognized by the anti-TAGE antibody are hereinafter referred to as TAGE.

## 3. Detection of Intracellular and Circulating TAGE

Different epitopes are recognized by the anti-TAGE antibody [51] and GA-derived AGE structures with a pyridinium ring, such as GLAP [52] and triosidines [53]. Neither structure has AGE-specific fluorescence or protein cross-links. We identified two compounds with a 1,4-dihydropyrazine ring that have fluorescence and cross-links to be TAGE candidate structures [PCT/JP2019/34195].

### 3.1. Slot Blot (SB) Analysis of Intracellular TAGE Levels

Total TAGE levels in cell extracts treated with reagents were assessed using a SB analysis with an anti-TAGE antibody. Cell extracts were prepared using lysis buffer (2 M thiourea, 7 M urea, 30 mM Tris, 4% 3-[(3-cholamido-propyl)-dimethyl-ammonio]-1-propane sulfonate, and protease inhibitor cocktail). Polyvinylidene difluoride membranes fixed with cell lysates using a SB apparatus were incubated with the anti-TAGE antibody and then with a horseradish peroxidase (HRP)-conjugated secondary antibody. A chemiluminescence reagent was used to detect immunoreactive proteins, and proteins were scanned using a chemiluminescence imaging system. TAGE levels in cell extracts were then calculated using a calibration curve for TAGE-bovine serum albumin (BSA).

### 3.2. Enzyme-Linked Immunosorbent Assay (ELISA) for TAGE Levels in Serum/Vitreous Fluid

A competitive ELISA with an anti-TAGE antibody was used to measure serum levels of TAGE, as previously described [51]. In brief, each well of a 96-well microtiter plate was coated with TAGE and incubated in a cold room overnight. Wells were washed three times with phosphate-buffered saline (PBS)-Tween-20 (PBS-Tween-20). Wells were then blocked by an incubation in a solution of PBS containing BSA. After washing with PBS-Tween-20, sera were added to each well as a competitor for the TAGE antibody, followed by an incubation. Wells were washed with PBS-Tween-20 and developed with an alkaline phosphatase-linked anti-rabbit IgG utilizing p-nitrophenyl phosphate as the colorimetric substrate. The following values were obtained for sensitivity and the intra- and inter-assay coefficients of variation: 0.01 U/mL, 6.2%, and 8.8%, respectively [55,56].

## 4. Cytotoxicity of TAGE

Many cell types that possess the pathways for the generation of GA from glucose and/or fructose may produce intracellular TAGE [9,10,11], and subsequently induce cell death. TAGE leak from cells and interact with RAGE to exert their effects on the surrounding cells. The TAGE-RAGE axis alters intracellular signaling, which in turn up-regulates the expression of RAGE that may contribute to LSRD.

### 4.1. TAGE Cytotoxicity in the Liver

The habitual and excessive consumption of SSB and processed foods that have a high content of sugar [57] and/or dietary AGEs (mainly Glu-/Fru-AGEs [58]) alters the metabolic system in hepatocytes, which leads to the excessive production of GA and, ultimately, the generation/accumulation of TAGE [9,10]. The strong cytotoxicity of TAGE causes intra-/extracellular damage in hepatocytes and hepatic stellate cells (HSC). Non-alcoholic fatty liver diseases (NAFLD) are currently recognized as the most common liver disorder. NAFLD is a spectrum of diseases that range from non-alcoholic fatty liver (NAFL) to NASH, which cause fibrosis, and may progress to cirrhosis, liver failure, and hepatocellular carcinoma (HCC).

#### 4.1.1. Intracellular TAGE and Hepatocyte Cell Death

A previous study reported the presence of detectable levels of TAGE in the hepatocytes of patients with NASH, and negligible levels in those of patients with NAFL [16]. The cytotoxicity of TAGE has been implicated in NASH-mediated dysfunctions in hepatocytes.

i. The accumulation of TAGE has been shown to induce damage in HCC cell lines (HepG2 and Hep3B) and primary cultured hepatocytes [30,31,32]. The intracellular accumulation of TAGE induced by GA increased the mRNA expression levels of C-reactive protein and decreased the chaperone activity of Hep3B cells [30]. The intracellular TAGE accumulation also induced necrotic-type cell death associated with TAGE-modified caspase-3 [31], and promoted the production of ROS in HepG2 cells and primary cultured hepatocytes [32].
ii. Heterogeneous nuclear ribonucleoprotein M (hnRNPM), an RNA-binding protein, was identified as a target protein for TAGE in Hep3B cells incubated in GA- or high fructose-containing media [59,60]. Furthermore, following the knockdown of hnRNPM, the up- or down-regulated expression of genes associated with extracellular exosome-containing extracellular spaces was observed [60].
iii. HCC cell lines and primary cultured cells have been used as in vitro models of NASH. However, they have several disadvantages, such as specialized characteristics by immortalization or limited growth potential. A decrease was noted in the viability of hepatocyte-like cells (HLCs), which differentiated from human induced pluripotent stem cells (hiPSCs) (hiPSC-HLCs), as TAGE accumulated in cells, which was consistent with previous findings on HCC cells and primary cultured hepatocytes [33]. In addition, the accumulation of TAGE up-regulated the expression of inflammation-related genes (i.e., interleukin (IL)-6, IL-8, and monocyte chemoattractant protein (MCP)-1) in hiPSC-HLCs. Experimental data from hiPSC-HLCs are considered to more accurately reflect the pathology of humans.

These studies provide a novel strategy for examining the involvement of hepatocyte toxicity due to the accumulation of TAGE in the pathogenesis of NASH.

#### 4.1.2. Effects of Extracellular TAGE on HSC

The activation of HSC by cytokines, such as transforming growth factor (TGF)-β1, tumor necrosis factor (TNF)-α, and platelet-derived growth factors, has been reported in patients with chronic liver damage. This is followed by their differentiation into myofibroblast-like cells and the secretion of a large amount of extracellular matrix (ECM) material, including collagen I [61]. In our previous study, we showed that TAGE induced the expression of genes and proteins related to fibrogenesis and inflammation, such as TGF-β1, α-smooth muscle actin, collagen type Iα2, and MCP-1, in the cultured human HSC cell line, LI90, via NADPH oxidase-derived ROS production [62]. Furthermore, apoptosis was detected in TGF-β1-treated LX-2 cells, a human HSC cell line, and this effect was significantly suppressed by TAGE co-treatment. Therefore, TGF-β1-induced apoptosis was inhibited by TAGE. Although no significant changes were observed in the mRNA expression levels of collagen I in each cell, the TGF-β1 and TAGE co-treatment significantly increased the secretion of the collagen I protein into the culture medium [63]. Collectively, these findings suggest that the total production of ECM molecules, such as collagen I, was promoted by TAGE through the inhibition of apoptosis in HSC activated by the TGF-β1 treatment. Therefore, liver fibrosis in patients with chronic hepatitis, such as NASH, may be aggravated by TAGE.

### 4.2. Cytotoxicity of TAGE in Skeletal Muscle

Patients with LSRD, including type 2 DM and NASH, are at an increased risk of sarcopenia [64,65,66]. Sarcopenia is a progressive and generalized skeletal muscle disorder that is characterized by the accelerated loss of muscle mass and function, which is associated with adverse outcomes, including the deterioration of activities of daily living and quality of life in addition to an increased risk of falls and mortality [64]. Myoblasts are precursor cells in skeletal muscle that differentiate to form myotubes and, ultimately, myofibers. Previous studies demonstrated that the death of or dysfunctions in myoblasts resulted in the loss of skeletal muscle [64,67,68]. Although patients with LSRD are at risk of sarcopenia, the mechanisms by which cell death is induced in myoblasts have not yet been elucidated in detail. We recently reported that the intracellular production of TAGE with a physiological concentration of GA strongly induced cell death in the mouse myoblast cell line C2C12 [34]. In contrast, we also reported that circulating TAGE levels increased in STAM mice, a NASH mouse model, which had steatohepatitis and fibrosis. Based on these findings, we investigated whether extracellular TAGE induced cell death in C2C12. The findings obtained showed that extracellular TAGE, at a level that was five-fold higher than the physiological concentration, did not induce cell death. Since extracellular TAGE were not cytotoxic at a physiological concentration, cell death in myoblasts only appears to be induced by intracellular TAGE.

### 4.3. Cytotoxicity of TAGE in Pancreatic Islet β-Cells (β-Cells)

A decrease in the number of β-cells, which produce and secrete insulin, contributes to the pathophysiology of DM [69]. The death of and dysfunctions in β-cells have been implicated in the development of glucotoxicity, lipotoxicity, and oxidative stress [70,71]. Autophagy protects β-cells against dysfunction and death in DM [70]. TAGE are cytotoxic and play a role in the pathogenesis of DM. Elevated serum TAGE levels have been reported in DM patients, and are cytotoxic. Intracellular TAGE have been shown to induce β-cell death, and may inhibit autophagy by reducing microtubule-associated protein 1 light chain 3 (LC3)-I, LC3-II, and p62, thereby suppressing their degradation. Although autophagy is generally associated with increases in the protein levels of LC3-II and decreases in those of LC3-I and p62, the levels of all of these proteins were reduced in DM. This pattern of reduced protein levels of LC3-I, LC3-II, and p62 is rare, and suggests the inhibition of autophagy by intracellular TAGE. However, a previous study demonstrated that physiological concentrations of extracellular TAGE were not cytotoxic [35]. Collectively, these findings will contribute to a more detailed understanding of the involvement of TAGE in the pathogenesis of DM.

### 4.4. Cytotoxicity of TAGE in Bone

Bone is remodeled through continuous resorption by osteoclasts and the replacement of old bone with new bone formed by osteoblasts. The differentiation and maturation of osteoblasts are essential for the formation of new bone, with the depletion of osteoblasts inducing osteoporosis. DM has been identified as one of the risk factors for osteoporotic fractures. Previous studies demonstrated that the risk of fractures was higher for DM patients [72,73]. Fractures significantly reduce quality of life and worsen life expectancy [74]. We recently reported GA-induced mouse osteoblastic cell death and detected TAGE modifications in several intracellular proteins. Moreover, a correlation was observed between decreases in the expression levels of both Runx2, an essential transcription factor for the differentiation of osteoblasts, and collagen and the accumulation of TAGE. The GA treatment also decreased normal protein levels of collagen in cells, suggesting that TAGE-induced modifications in collagen resulted in the formation of an abnormal structure. These findings revealed for the first time the cytotoxic effects of GA and TAGE against osteoblasts, their suppression of osteoblastic differentiation, and their reduction of normal collagen levels [36]. These findings provide evidence for the involvement of TAGE in the pathogenesis of osteoporotic fractures associated with DM.

### 4.5. Cytotoxicity of TAGE in the Heart

CVD is induced by the death of and dysfunctions in cardiomyocytes. TAGE have been suggested to play a role in the increased risk of CVD in patients with DM. Cardiac fibroblasts are activated and differentiate by specific signals from cardiomyocytes, and play a critical role in the induction of injury responses.

#### 4.5.1. Intracellular TAGE and Cardiomyocyte Death

We recently revealed that intracellular TAGE decreased beating rates and induced cell death in cardiomyocytes without myocardial ischemia as well as diabetic adverse cardiac remodeling [37], and also reported elevated serum TAGE levels in patients with myocardial ischemia [9,10]. Furthermore, we indicated the inhibition of autophagy by intracellular TAGE [37]. Although previous studies demonstrated that intracellular TAGE were cytotoxic in cell lines of various organs [30,31,33,34,35,36,37,38,39,42], our findings showed time-dependent decreases in the expression levels of LC3-II/LC3-I in cardiomyocytes treated with GA [37]. TAGE-modified proteins in cardiomyocytes have yet to be identified; however, TAGE have been suggested to alter the proteins involved in the regulation of autophagy-related pathways, such as those contributing to the generation of LC3-II. The intracellular production of TAGE may occur in human and rat cardiomyocytes and directly induce cell damage, thereby contributing to CVD.

#### 4.5.2. Intracellular TAGE and Human Cardiac Fibroblast (HCF) Cell Death

Cardiac fibroblasts are activated and differentiate into cardiac myofibroblasts by specific signals, such as TGF-β from cardiomyocytes; they produce and secrete proteins associated with the ECM, such as collagen, to protect cardiac tissues; they also play a critical role in the induction of injury responses. We treated HCF with GA, which resulted in the production of intracellular TAGE and, ultimately, cell death [38]. In contrast, extracellular levels of TAGE that were approximately 10- to 30-fold higher than physiological concentrations did not induce cell death in HCF [38]. Only intracellular TAGE induced cell death in HCF under physiological conditions, and may directly suppress heart repair or injury. The activation of cardiac fibroblasts is essential for repair and remodeling after the induction of cell death in cardiomyocytes by intracellular TAGE; however, intracellular TAGE in these cells may impair their functions, which suppresses the fibrotic healing response and potentially results in heart dysfunction.

#### 4.5.3. Extracellular TAGE and Cardiomyocytes and HCF

Extracellular TAGE in the vascular system have been implicated in the development of CVD, and relationships have been reported between serum TAGE levels and the risk factors for CVD [20,21,22,55,75]. We previously reported that the activation of the extracellular TAGE-RAGE axis led to the intracellular production of ROS and activation of nuclear factor (NF)-κB in vascular wall cells, which have been suggested to up-regulate the expression of various genes related to atherosclerosis and inflammation, and promote the onset and progression of CVD in DM [15]. Cardiomyocytes and cardiac fibroblasts both express RAGE, and hence a cytotoxic response may be induced in these cells by the TAGE-RAGE axis. However, extracellular TAGE did not directly inhibit the pulsation of cardiomyocytes nor induce cell death in cardiomyocytes or cardiac fibroblasts [38].

### 4.6. Cytotoxicity of TAGE in the Brain

DM has recently been proposed as a risk factor for the onset and progression of AD [76,77]. AD is characterized by the accumulation of senile plaques and neurofibrillary tangles (NFT), which mainly comprise amyloid β (Aβ) protein and tau protein, respectively [76,78]. A previous study reported that TAGE mainly localized in the cytosol of neurons in the hippocampus and parahippocampal gyrus in AD brains [24]. We also demonstrated that TAGE were stronger neurotoxins than AGEs, such as Glu-AGEs and CML, in neurons [23,79].

#### 4.6.1. Intracellular TAGE and Neuronal Cells

In our previous study, we showed the following:

i. The accumulation of intracellular TAGE in GA-treated human neuroblastoma SH-SY5Y cells, which resulted in cell death [39];
ii. A reduction in the activity of glyceraldehyde-3-phosphate dehydrogenase (GAPDH) and the inhibition of glycolysis in GA-treated neuronal cells [39];
iii. A reduction in the activity of GAPDH in AD patients [80]. We also demonstrated the involvement of GAPDH in apoptosis in neurodegenerative disorders [81].

We found that TAGE induced similar alterations to those observed in AD. GA induced dysfunctional neurite outgrowth via TAGE-β-tubulin aggregation, which resulted in the TAGE-dependent abnormal aggregation of β-tubulin and tau phosphorylation in SH-SY5Y cells [40]. These findings indicated that the formation of TAGE-β-tubulin contributed to the production of paired helical filaments, one of the components of NFT. More recently, we showed that AGE inhibitors (i.e., aminoguanidine and pyridoxamine) suppressed the formation of TAGE-β-tubulin, mitigated the GA-induced inhibition of neurite outgrowth, and reduced GA-mediated increases in tau phosphorylation levels [41]. Glial intracellular TAGE accumulation may induce cell death in primary cultured astrocytes and contribute to the disruption of the blood-brain barrier (unpublished data). Therefore, intracellular TAGE may be causative agents for the onset and progression of AD.

#### 4.6.2. Extracellular TAGE on Neuronal Cells

We previously demonstrated the stronger neurotoxicity of TAGE in a neuronal primary culture system than Glu-AGEs, MGO-AGEs, and CML, which are AGEs that have been examined in detail [23,79]. Moreover, the addition of an anti-TAGE antibody completely mitigated the neurotoxicity of serum AGEs in HD-DN patients, whereas antibodies of other types of AGEs or CML did not [23,79].

#### 4.6.3. Extracellular TAGE on Brain Vascular Endothelial Cells (EC)

The vascular endothelium, a monolayer of vascular EC, functions as a barrier that regulates the permeation of blood components through vessel walls. We previously demonstrated that TAGE increased tissue factor (TF), a major regulator of normal hemostasis and pathological thrombosis, the expression of which is associated with the overproduction of ROS [82]. We also demonstrated that brain microvascular EC were more susceptible than aortic vascular EC to TAGE-enhanced permeability, which was dependent on VEGF expression induced by the TAGE-RAGE-ROS axis [83]. We recently reported that TAGE induced enhancements in vascular permeability through the disruption of adherens junctions and tight junctions via complex signaling, including ROS and non-ROS pathways. Ras guanyl nucleotide releasing protein 2 (RasGRP2), which activates small G proteins, prevented the disruption of adherens junctions, which inhibited vascular hyperpermeability. These findings suggest that the essential role of RasGRP2 is as a protective factor against vascular permeability, and will contribute to the development of novel therapeutic strategies for TAGE-induced LSRD [84]. Therefore, vascular permeability is increased in the brain of DM patients and the transfer of serum TAGE into the brain is promoted.

### 4.7. Cytotoxicity of TAGE in Cancer

TAGE appear to bind to RAGE, leading to the release of pro-inflammatory cytokines, increased cellular damage, and the higher expression of VEGF in cancer cells [15,49]. Despite promising experimental evidence [26,27], there were no previous observational studies on circulating TAGE and the risk of death among cancer survivors. Two cell culture studies suggested that TAGE enhanced the migration and invasion of melanoma [26] and lung cancer cells [27], and thus may contribute to the progression of cancer.

#### 4.7.1. Melanoma

In our previous study, we showed that the growth and migration of human melanoma G361 cells were stimulated by TAGE [26]. Furthermore, neutralizing antibodies raised against RAGE prevented the formation of tumors by melanoma cell xenografts in athymic mice. Survival rates were higher in tumor-bearing mice, and the anti-RAGE antibody suppressed spontaneous pulmonary metastases of melanoma. An examination of the beds of human melanoma tumors and normal skin revealed the presence of detectable and negligible levels of TAGE, respectively.

#### 4.7.2. Lung Cancer

We also investigated the mechanisms by which TAGE increase the malignancy of cancer using the lung cancer cell line A549. The findings obtained showed that (i) cell proliferation was suppressed by the addition of TAGE, (ii) TAGE significantly increased cell migration and infiltration ability, and (iii) extracellular TAGE induced the production of ROS via RAGE. Rac1 was activated by the production of ROS, which enhance cell migration ability, while the activation of matrix metalloproteinase 2 promoted infiltration ability [27].

#### 4.7.3. Pancreatic Cancer

PANC-1 cells were treated with GA, which induced the production of intracellular TAGE and cell death. The high-molecular-weight complexes of heat shock proteins (i.e., heat shock protein (HSP)90β, HSP70, and HSP27) were produced after the GA treatment in a dose-dependent manner. We considered high-molecular-weight complexes to be dimers and trimers with TAGE-mediated aggregation. Extracellular TAGE promoted the proliferation of PANC-1 cells [42]. Therefore, although intracellular TAGE induce pancreatic cancer cell death, their secretion and release may promote the proliferation of other pancreatic cancer cells.

#### 4.7.4. Colorectal Cancer (CRC)

TAGE possess pro-inflammatory and pro-oxidative properties, which may contribute to the progression of CRC and poor survival. The TAGE-RAGE axis has been suggested to trigger multiple signaling pathways (e.g., NF-κB and phosphatidylinositol 3 kinase/protein kinase B), resulting in increases in the hyperproliferation and/or metastatic potential of CRC cells and CRC progression. Furthermore, chronic hyperglycemia may facilitate the generation of AGEs, and has been associated with CRC progression via various metabolic pathways, including the insulin growth factor pathway. These findings suggest that investigations on TAGE in CRC survivors will provide novel insights into the prevention of CRC progression and improvements in CRC survival-related outcomes [29].

In other words, the TAGE-RAGE axis may increase malignancy from the growth stage to the metastasis/infiltration stage of cancer cells by enhancing the production of ROS in cancer cells.

### 4.8. Limitation

The TAGE stress and oxidative stress have been clarified in the research using hepatocytes [32], but the progress of the research involving the target molecule is still forthcoming. The relationship between DM and oxidative stress is important in other cells, including erythrocytes, and findings on various AGEs and active oxygen have been reported [43,85]. This review focuses on the findings related to TAGE, in order to distinguish it from the findings of other AGEs and ALEs.

### 4.9. Brief Summary and Perspectives

The effects of AGEs have been attributed to their extracellular binding to RAGE or their accumulation in numerous tissues; however, further studies are warranted to elucidate the impact of intracellular TAGE. In vitro intracellular TAGE molecules have been shown to exert cytotoxic effects in hepatocytes [30,31,33], myoblasts [34], β-cells [35], osteoblasts [36], cardiomyocytes [37], cardiac fibroblasts [38], neuroblastoma cells [39], and pancreatic ductal cells [42]. These molecules are intermediates of the abnormal metabolism of glucose and fructose in the presence of excess GA. The involvement of TAGE in apoptotic and/or necrotic events, and hence cell death and tissue damage, has been suggested.

## 5. Clinical Relevance of Circulating TAGE Levels and LSRD

The accumulation of intracellular TAGE contributes to a number of cellular disorders, and their leakage into the extracellular space increases circulating TAGE levels. Therefore, circulating TAGE levels have potential as novel biomarkers for predicting the onset and progression of LSRD (Table 1 and Table 2).

### 5.1. Healthy Population (Apparently Healthy/General Population)

Endothelial progenitor cells (EPC) maintain the structure and function of the endothelium, and thus facilitate angiogenesis and vascular repair. High serum levels of TAGE independently correlated with decreases in the number and migratory activity of circulating EPC in healthy volunteers with normal blood test values [86], suggesting TAGE-induced impairments in the repair of EC. We previously demonstrated that serum levels of TAGE, but not HbA1c, Glu-AGEs, or CML, were associated with thrombogenic markers, including plasminogen activator inhibitor-1 and fibrinogen [75,87], in the general population. Furthermore, correlations were reported between flow-mediated vasodilation and serum levels of TAGE and the soluble form of RAGE (sRAGE), as well as the ratio of TAGE to sRAGE in health examinations [88]. In healthy volunteers administered collagen tripeptide, an inhibitor of the generation of TAGE, the cardiac-ankle vascular index, an index of blood vessel stiffness, was shown to decrease with reductions in serum levels of TAGE [22].

### 5.2. Non-Diabetic General Population (Outpatients)

We previously revealed that serum TAGE levels independently correlated with serum levels of sRAGE [89], low-density lipoprotein cholesterol [90], serum pigment epithelium-derived factor, markers of insulin resistance (IR) [91], the homeostatic model assessment of IR (HOMA-IR) index [92], adiponectin (inversely) [93,94], and dipeptidyl peptidase-4 (DPP-4) [95] in a non-diabetic general population. We also found that HOMA-IR was independently associated with high serum levels of TAGE and low testosterone [96]. Furthermore, TAGE and sRAGE levels correlated with each other, while TAGE and high mobility group box 1 were independently associated with asymmetric dimethylarginine (ADMA) in non-diabetic chronic kidney disease (CKD) patients [97]. Atorvastatin, a 3-hydroxy-3-methyl-glutaryl-CoA reductase inhibitor, may decrease proteinuria in non-diabetic CKD with dyslipidemia partly by reducing serum levels of TAGE [108]. The calcium channel blocker, azelnidipine, but not amlodipine, decreased serum levels of TAGE, sRAGE, and proteinuria [109]. Azelnidipine may exert renoprotective effects in non-diabetic hypertensive CKD patients via its unique inhibition of the TAGE-RAGE axis.

### 5.3. Diabetic Outpatients

Serum TAGE levels were previously reported to be higher in diabetic patients than in healthy controls [13]. A correlation was observed between serum TAGE levels and sRAGE, which may reflect tissue RAGE expression, in non-diabetic and diabetic subjects [89,98,99], indicating their potential as a biomarker for activation of the TAGE-RAGE axis. Correlations were also detected between serum TAGE levels and inflammatory biomarkers, such as MCP-1 [13], and the soluble form of vascular cell adhesion molecule-1 [99] in type 2 DM. We demonstrated significant reductions in serum TAGE levels in DM patients treated with acarbose for 12 weeks [14]. We also found that serum TAGE levels were significantly reduced by a DPP-4 inhibitor [110], sulfonyl urea [111], and insulin [112], and these decreases were associated with reductions in the levels of biomarkers of organ damage in DM and CKD patients. Furthermore, we showed decreases in serum TAGE levels in T2DM patients treated with atorvastatin [113]. Collectively, these findings indicate the potential of serum TAGE levels as a new biomarker for the early diagnosis of LSRD or in assessments of the effectiveness of therapeutic strategies to prevent/treat the onset and progression of LSRD regardless of the presence/absence of DM.

### 5.4. CVD/Heart Failure

We previously reported the following:

i. Serum levels of TAGE, but not HbA1c or CML, independently correlated with vascular inflammation in outpatients using [^18^F] fluorodeoxyglucose-positron emission tomography [55];
ii. Among pre-DM patients, circulating levels of TAGE were significantly higher in the high mean amplitude of the glycemic excursions (MAGE) group than in the low MAGE group [21]. TAGE and medication for hypertension were independently associated with area of visceral adipose tissues, whereas medication for TAGE, DM, intima-media thickness, and PEDF were independent correlates of subcutaneous adipose tissue areas [100].

TAGE were also independently associated with log trimethylamine. The TAGE to sRAGE ratio correlated with log trimethylamine N-oxide, a marker of cardiometabolic disorders [101]. More recently, the levels of TAGE and TNF-α showed close associations with left ventricular ejection fraction and brain natriuretic peptide values in patients with diabetic adverse cardiac remodeling [102]. TAGE and TNF-α may play a pathological role in the development of diabetic adverse cardiac remodeling. We previously reported that elevated baseline TAGE levels were associated with the progression of plaques in an assessment of pitavastatin and atorvastatin in an acute coronary syndrome trial (The JAPAN-ACS Sub-study) [20]. High baseline TAGE levels were associated with the progression of plaques in the JAPAN-ACS trial. Statin therapy was initiated early after the onset reduced TAGE levels and appeared to exert cardioprotective effects in patients with acute myocardial infarction [114].

### 5.5. Infertility

We previously reported age-related decreases in the number of oocytes collected and pregnancy rates; ongoing pregnancy rates were also reduced in younger women with elevated serum TAGE levels [56]. Furthermore, correlations were observed between serum TAGE levels and follicle development, fertilization, embryo development, and pregnancy in assisted reproductive technologies (ART), indicating the novelty and utility of the accumulation of TAGE as an indicator of poor responders that is independent of age and day-3 follicle-stimulating hormone levels [56]. Among non-pregnant poor responders who were administered the DPP-4 inhibitor, sitagliptin, and underwent ART, the amelioration of ovarian dysfunction and significant increases in ongoing pregnancy rates were observed in those whose serum TAGE levels were decreased by sitagliptin [115]. A prospective randomized open-label controlled trial was conducted on women undergoing ART with or without Hishi (a safe herbal medicine that markedly inhibits the formation of AGEs in vitro) extract. The cumulative live birth rate was significantly higher in Hishi patients than in controls (47% vs. 16%). The live birth rate per embryo transfer, including fresh and cryopreserved, was significantly higher with Hishi (28% vs. 10%). Hishi significantly enhanced oocyte developmental potential, improved endometrial receptivity in natural cycles, and decreased serum and follicular fluid levels of TAGE [116]. Collectively, these findings indicate the potential of serum TAGE levels as a biomarker in evaluations of the prevention, early diagnosis, and treatment of ovarian dysfunction.

### 5.6. NASH

NAFLD is a spectrum of diseases that ranges from NAFL to NASH, and is one of the most common causes of hepatic disease. We examined serum levels of AGEs (TAGE, Glu-AGEs, and CML) in NASH patients with no evidence of liver cirrhosis, in NAFL patients, and in healthy controls [16]. The accumulation of TAGE in both tissue and serum was higher in NASH patients than in NAFL patients and healthy controls. A positive correlation was observed between serum TAGE levels and HOMA-IR, while an inverse association was noted between serum levels of TAGE and adiponectin levels. TAGE were present at detectable levels in the hepatocytes of NASH patients, but at negligible levels in those of NAFL patients, and Glu-AGE or CML levels did not significantly differ between these groups [16]. We previously reported that serum levels of TAGE in NASH patients with dyslipidemia were reduced by a treatment with atorvastatin [17]. A 6-month treatment with atorvastatin significantly decreased the activities of liver alanine aminotransferase and γ-glutamyl transpeptidase (γ-GTP) in all patients. Moreover, increases were observed in plasma adiponectin levels and decreases in plasma TNF-α levels in NASH and NAFL patients, while serum levels of TAGE significantly decreased. Collectively, these findings suggest the potential of serum levels of TAGE, but not Glu-AGEs or CML, as a biomarker for predicting the progression of NASH.

### 5.7. Cancer (Non-B or Non-C (NBNC)-HCC and CRC)

#### 5.7.1. NBNC-HCC

We previously reported that serum levels of TAGE were significantly higher in NBNC-HCC patients than in NASH patients without HCC and in control subjects [18]. A multiple regression analysis revealed that age and the levels of γ-GTP and high-density lipoprotein cholesterol (inversely) independently correlated with TAGE levels. Therefore, TAGE appear to play a role in the pathogenesis of NBNC-HCC, and thus have potential as biomarkers for discriminating NBNC-HCC from NASH.

#### 5.7.2. CRC

We conducted a large nested case-control study within the European Prospective Investigation into Cancer and Nutrition (EPIC) cohort, in order to investigate the relationship between TAGE and the risk of CRC [29]. A correlation was not observed between TAGE concentrations and the risk of CRC. However, in a sub-group analysis, a positive correlation was noted between TAGE and the risk of rectal cancer [29]. Additionally, the risk of rectal cancer was higher in individuals who consumed alcohol. Substantial clinical evidence supports a role for TAGE in the pathophysiology of DM and other LSRD [9,10]; however, observational studies have not yet been conducted among cancer survivors. During a mean follow-up of 48 months, TAGE positively correlated with all-cause and CRC-specific mortality among patients with CRC. A stronger positive association between TAGE and mortality was observed among distal colon cancer cases (unpublished data). Therefore, our findings support the direct association between serum TAGE and CRC-specific and all-cause mortality among CRC patients.

### 5.8. Schizophrenia and AD

We previously reported significant increases and decreases in TAGE and sRAGE levels, respectively, in patients with acute schizophrenia, which remained stable over the clinical course [103]. Furthermore, patients with schizophrenia had a higher TAGE to sRAGE ratio than healthy control subjects. These findings confirmed the utility of TAGE in addition to the TAGE to sRAGE ratio as diagnostic markers of schizophrenia. However, in neuropathological studies on schizophrenia without prominent neurodegeneration, schizophrenia is considered to be a functional disease, indicating the different roles of increases in TAGE levels in schizophrenia and neurodegenerative diseases. Previous studies demonstrated that AD patients had significantly lower Aβ42 levels and significantly higher total tau and p-tauT181 levels in cerebrospinal fluid (CSF) than age-matched healthy elderly controls [120,121]. Furthermore, other AD biomarkers in the CSF, such as VEGF [122] and TGF-β1 [123], were elevated in AD patients. In our recent study, we showed the following effects of the intracellular production of TAGE: reductions in the level of Aβ42 and increases in the levels of total tau and p-tauT181 in culture media, as well as increases in the intracellular levels of total tau, p-tauT181, VEGF, and TGF-β in human neuroblastoma SH-SY5Y cells [39]. Therefore, CSF and/or serum levels of TAGE have potential as a useful biomarker for the early detection of AD [124].

### 5.9. Other Diseases

ADMA levels were significantly elevated in patients with septic shock, and serum TAGE levels were identified as independent determinants of ADMA [104]. Serum TAGE levels were significantly higher for each etiology of uveitis (leukocyte antigen (HLA)-B27, Vogt–Koyanagi–Harada (VKH) disease, Bechet’s disease, and sarcoidosis) than in healthy controls [105]. In DM patients, serum levels of TAGE increased as the stages of retinopathy and nephropathy developed [106]. The administration of AST-120 significantly decreased serum levels of TAGE in non-diabetic chronic renal failure patients [117]. The vasculoprotective properties of L-carnitine in HD patients may be partly attributed to its inhibitory effects on TAGE [118]. We previously reported a positive correlation between vitreous levels of TAGE and VEGF in patients with diabetic retinopathy [107] and in those with diabetic retinopathy treated with photocoagulation [119].

### 5.10. Brief Summary and Perspectives

High serum levels of TAGE predict the onset and progression of various LSRD, even in healthy subjects with normal blood test values. Therefore, the findings of our series of studies indicate the potential of serum levels of TAGE as a useful biomarker for the prevention/early diagnosis of LSRD and in evaluations of the efficacy of treatments for LSRD. The measurement of TAGE levels via specific competitive ELISA [9] may facilitate the identification of high-risk patients and provide important information for treatment decision-making in the future.

## 6. Conclusions and Perspectives

**Figure 3:**
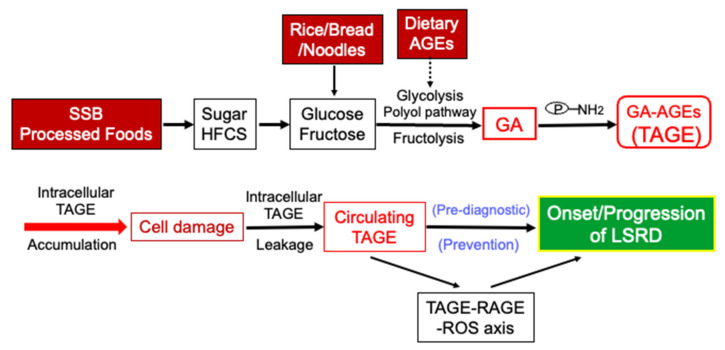
The onset and progression of lifestyle-related diseases (LSRD) are associated with the habitual excessive intake of sugars and/or dietary AGEs. TAGE are produced from the metabolite of glucose, the main component of rice, bread, and noodles, as well as the metabolites of sugars (sucrose and HFCS) added to beverages and processed foods. Fluctuations in TAGE levels in the human body are closely associated with dietary habits. The chronic intake of excessive amounts of SSB, processed foods, and/or rice/bread/noodles, which is characteristic of the modern daily diet, increases the cellular levels of the sugar metabolite glyceraldehyde (GA), which promotes the production of TAGE from intracellular proteins. Elevated levels of TAGE damage cells, which results in the leakage of TAGE into the circulation, thereby increasing circulating TAGE levels. Moreover, the habitual and excessive consumption of excessive dietary AGEs (mainly Glu-/Fru-AGEs) promotes the accumulation of TAGE and up-regulates the expression of RAGE, resulting in TAGE-RAGE interactions. Activation of the TAGE-RAGE axis leads to the production of ROS, which up-regulate the expression of RAGE and promote the generation of TAGE, which have been implicated in the onset and progression of LSRD. SSB: sugar-sweetened beverages; HFCS: high-fructose corn syrup; AGEs: advanced glycation end-products; GA: glyceraldehyde; TAGE: toxic AGEs; RAGE: receptor for AGEs; ROS: reactive oxygen species; LSRD: lifestyle-related diseases; P-NH_2_: free amino residue of protein.

The habitual and excessive consumption of SSB, processed foods, and/or rice/bread/noodles, which are characteristic of the modern daily diet, have been shown to elevate cellular levels of GA, which, in turn, promotes the production of TAGE from intracellular proteins [9,10,11]. Elevated levels of TAGE damage cells, which results in the leakage of TAGE into the circulation, thereby elevating circulating TAGE levels. Moreover, the habitual and excessive consumption of dietary AGEs (mainly Glu-/Fru-AGEs) promotes the accumulation of TAGE and up-regulates the expression of RAGE, resulting in TAGE-RAGE interactions. Activation of the TAGE-RAGE axis leads to the production of ROS, which up-regulate the expression of RAGE and promote the generation of TAGE, which have been implicated in the onset and progression of LSRD (Figure 3). Collectively, our findings indicate that pre-diagnostic circulating TAGE levels are positively associated with LSRD. The novel concept of the “TAGE theory” is expected to open new perspectives for research into numerous diseases.

## Figures and Tables

**Table 1:** Clinical relevance of circulating TAGE levels.

Subjects	Main Findings	Ref.
Apparently Healthy	Serum TAGE levels independently correlated with decreases in the number and migratory activity of circulating EPC in apparently healthy subjects.	[86]
Health Examination (General Population)	Serum TAGE levels were independent determinants of PAI-1 in the general population.	[87]
	A positive relationship was observed between fibrinogen levels and serum TAGE levels. TAGE-associated thrombogenic abnormalities may be involved in atherogenesis.	[75]
	Flow-mediated vasodilation correlated with serum levels of TAGE and sRAGE, and the ratio of TAGE to sRAGE.	[88]
Non-Diabetic General Population (Outpatients)	Serum sRAGE levels were positively associated with serum TAGE levels.	[89]
	LDL-C levels were independent determinants of serum TAGE levels.	[90]
	Serum TAGE levels independently correlated with serum PEDF levels. PEDF levels may be elevated in response to TAGE levels as a counter system against TAGE-elicited tissue damage.	[91]
	Serum TAGE levels independently correlated with the HOMA-IR index, suggesting that TAGE play a pathological role in insulin resistance.	[92]
	Adiponectin was inversely associated with the ratio of serum levels of TAGE to sRAGE and vascular inflammation.	[93]
	Serum levels of apoB48 were correlated with TAGE, PEDF, and adiponectin (inversely).	[94]
	Serum DPP-4 levels were independently associated with various metabolic parameters. TAGE may up-regulate cellular DPP-4 expression and subsequently increase circulating levels of DPP-4.	[95]
Non-Diabetic Men	HOMA-IR was independently associated with high serum levels of TAGE and low testosterone.	[96]
Non-Diabetic Chronic Kidney Disease (CKD)	Serum TAGE and sRAGE levels correlated with each other, and TAGE and HMGB-1 were independently associated with ADMA.	[97]
Type 2 DM	Serum sRAGE levels were positively associated with serum TAGE levels.	[98]
	Serum sRAGE levels were positively associated with serum TAGE and sVCAM-1 levels.	[99]
	Serum levels of TAGE and sRAGE were independent determinants of serum MCP-1 levels.	[13]
Cardiovascular Disease (CVD)/Heart Failure	Serum TAGE levels were independently associated with vascular inflammation evaluated by FDG-PET, suggesting that serum TAGE levels are a biomarker that reflects vascular inflammation within an area of atherosclerosis.	[55]
	Diurnal glycemic fluctuations (GF) were associated with the severity of CAD, even in prediabetic patients. GF and TAGE levels may play a pathological role in the progression of CAD.	[21]
	TAGE and medication for hypertension were independently associated with area of visceral adipose tissues, whereas medication for TAGE, DM, IMT, and PEDF were independent correlates of subcutaneous adipose tissue areas.	[100]
	Serum TAGE levels were independently associated with log TMA. The TAGE to sRAGE ratio correlated with log TMAO, a marker of cardiometabolic disorders.	[101]
	Serum TAGE and TNF-α levels were associated with LVEF and BNP values in patients with diabetic adverse cardiac remodeling.	[102]
Infertile Women	Serum TAGE levels correlated with poor follicular and embryonic development and a lower likelihood of ongoing pregnancy.	[56]
Non-Alcoholic Steatohepatitis (NASH)	Serum TAGE levels were significantly higher in NASH patients than in NAFL or healthy controls. Moreover, TAGE inversely correlated with adiponectin.	[16]
Non-B or Non-C-Hepatocellular Carcinoma	Serum TAGE levels were significantly higher in NBNC-HCC patients than in NASH and control subjects.	[18]
Colorectal Cancer	Serum TAGE levels were not associated with the risk of colon cancer, but showed a positive association with the risk of rectal cancer.	[29]
Schizophrenia	Serum TAGE levels were significantly higher, and sRAGE levels were significantly lower in patients with acute schizophrenia than in healthy controls.	[103]
Septic Shock Patients	Serum ADMA levels were significantly elevated in patients with septic shock, and serum TAGE levels were independent determinants of ADMA.	[104]
Autoimmune Uveoretinitis	Serum TAGE levels were significantly higher for each etiology of uveitis (HLA-B27, VKH disease, Bechet’s disease, and sarcoidosis) than in healthy controls.	[105]
Diabetic Retinopathy/Nephropathy	In diabetic patients, serum TAGE levels increased as the stages of retinopathy and nephropathy developed.	[106]
Diabetic Retinopathy	A positive correlation was observed between vitreous levels of TAGE and VEGF in patients with diabetic retinopathy.	[107]

TAGE, toxic AGEs; EPC, endothelial progenitor cells; PAI-1, plasminogen activator inhibitor-1; sRAGE, soluble form of receptor for AGEs; LDL-c, low-density lipoprotein cholesterol; PEDF, pigment epithelium-derived factor; HOME-IR, homeostatic model assessment of insulin resistance; DPP-4, dipeptidyl peptidase-4; HMGB-1, high mobility group box 1; ADMA, asymmetric dimethylarginine; sVCAM-1, soluble form of vascular cell adhesion molecule 1; MCP-1, monocyte chemoattractant protein 1; FDG-PET, fluorodeoxyglucose-positron emission tomography; IMT, intima-media thickness; TMA, trimethylamine; TMAO, trimethylamine N-oxide; CAD, coronary artery disease; TNF-α, tumor necrosis factor-α; LVEF, left ventricular ejection fraction; BNP, brain natriuretic peptide; NAFL, non-alcoholic fatty liver; NBNC-HCC, non-B or non-C-hepatocellular carcinoma; HLA, leukocyte antigen; VKH, Vogt–Koyanagi–Harada; VEGF, vascular endothelial growth factor.

**Table 2:** Changes in circulating TAGE levels with treatment.

Subjects	Therapeutic Agents	Correlation Factor	Ref.
Healthy Humans	Collagen tripeptide (CTP)	A significant reduction in serum TAGE levels was observed in all subjects and in the high-risk group after the CTP treatment.	[22]
Non-Diabetic CKD	Statin (Atorvastatin)	Atorvastatin may attenuate proteinuria in non-diabetic CKD with dyslipidemia partly by reducing serum TAGE levels.	[108]
Non-Diabetic Hypertensive CKD	Calcium channel blocker (Azelnidipine)	A treatment with azelnidipine decreased serum levels of TAGE, sRAGE, and proteinuria.	[109]
Type 2 DM	α-Glucosidase inhibitor (Acarbose)	A treatment with acarbose significantly decreased serum TAGE and free fatty acid levels.	[14]
	DPP-4 inhibitor (Alogliptin)	Serum TAGE levels were only reduced in patients with baseline TAGE >7 U/mL after a treatment with alogliptin.	[110]
	Sulfonyl urea (Glimepiride)	Glimepiride may repair tissue damage by decreasing serum TAGE levels.	[111]
	Insulin (Glulisine)	Switching to multiple daily injection therapy with glulisine decreased serum levels of TAGE and sRAGE.	[112]
	Statin (Atorvastatin)	Atorvastatin decreased serum TAGE levels in hypercholesterolaemic T2DM patients.	[113]
JAPAN-ACS Sub-Study	Statin (Pitavastatin/Atorvastatin)	Serum TAGE levels significantly decreased with statin therapy, whereas sRAGE levels did not change.	[20]
SAMIT (Statin for Acute Myocardial Infarction Trial)	Statin (Atorvastatin)	Statin therapy initiated early after the onset reduced serum TAGE levels, and may exert cardioprotective effects in patients with AMI.	[114]
Infertile Women	DPP-4 inhibitor (Sitagliptin)	Ovarian dysfunction was attenuated, and ongoing pregnancy rates were significantly increased in the group treated with sitagliptin, which decreased serum TAGE levels.	[115]
	Hishi (Trapa bispinosa Roxb.) extract	Hishi lowered serum TAGE levels and increased live births in older patients with ART.	[116]
NASH with Dyslipidemia	Statin (Atorvastatin)	Atorvastatin decreased serum TAGE levels in NASH patients with dyslipidemia.	[17]
Non-Diabetic CRF Patients	Oral adsorbent (AST-120/Kremedin)	The administration of AST-120 significantly decreased serum TAGE levels in non-diabetic CRF patients.	[117]
Hemodialysis (HD) Patients	L-Carnitine	The vasculoprotective properties of L-carnitine in HD patients may be partly attributed to its inhibitory effects on TAGE.	[118]
Diabetic Retinopathy	Photocoagulation	A positive correlation was observed between vitreous levels of TAGE and VEGF in patients with diabetic retinopathy sufficiently treated with photocoagulation.	[119]

CKD, chronic kidney disease; T2DM, type 2 diabetes mellitus; JAPAN-ACS, Japan assessment of pitavastatin and atorvastatin in acute coronary syndrome; AMI, acute myocardial infarction; ART, assisted reproductive technologies; CRF, chronic renal failure; VEGF, vascular endothelial growth factor.

## Data Availability

Data are contained within the article.
